# Pharmacological activation of cGMP signaling promotes astrocyte remodeling and enrichment of perivascular GLUT1 in the murine retina

**DOI:** 10.3389/fncel.2026.1870679

**Published:** 2026-07-02

**Authors:** Kristin L. Clark, Shane Mecca, Elio Almaoui, Olivia L. Bossardet, Joseph M. Holden, David J. Calkins, Lauren K. Wareham

**Affiliations:** 1 Vanderbilt Eye Institute, Vanderbilt University Medical Center, Nashville, TN, United States; 2 School of Medicine, University of Miami, Miami, FL, United States

**Keywords:** astrocyte, GLUT1, neurovascular coupling, neurovascular unit, perivascular endfeet, retina, retinal ganglion cell

## Abstract

Astrocytes regulate metabolic exchange between the vasculature and neurons in the central nervous system (CNS). In the retina of the eye, also a component of the CNS, astrocyte endfeet couple to vascular elements to mediate glucose uptake via the glucose transporter GLUT1 and shuttle metabolic resources to the axons of retinal ganglion cells (RGCs), which provide visual input to the brain. In addition to its own transcriptional regulation, GLUT1 is also modulated by signaling pathways that influence its subcellular localization at the astrocyte-vascular interface. For example, cyclic guanosine monophosphate (cGMP) signaling, a key regulator of vascular tone, is associated with changes in retinal astrocyte morphology and is implicated in age-related loss of RGCs, suggesting a potential role in astrocyte function within the neurovascular unit. Here, we tested this possibility directly by investigating the effects of 8-Br-cGMP on astrocyte morphology, vascular interactions, and GLUT1 localization in the retina. Using a transgenic mouse that allows resolution of individual astrocytes in great detail across the retina (the G-MORF mouse), we show that acute elevation of 8-Br-cGMP increases astrocyte coverage area without altering overall vascular structure. 8-Br-cGMP treatment was also associated with enhanced astrocyte-vascular association, reflected by increased endfoot coverage of blood vessels and altered scaling of astrocyte contact with vessel size. Treatment to increase cGMP signaling promoted redistribution of GLUT1 to astrocyte perivascular endfeet without changing overall levels of GLUT1. Together, these findings indicate that increased cGMP signaling induces coordinated structural and molecular remodeling of astrocytes at the vascular interface. These results provide important insight into how cyclic nucleotide signaling pathways may regulate astrocyte organization in the retina and suggests a potential role for cGMP in modulating astrocyte-vascular interactions within the neurovascular unit.

## 1 Introduction

Retinal ganglion cells (RGCs) sustain their high metabolic demand through uptake of glucose via membrane-localized glucose transporters GLUT1 and GLUT3 (Tabernero et al., 2006; Holoman et al., 2021). While RGCs are capable of direct glucose uptake, efficient metabolic support across the retina depends on coordinated neurovascular coupling mediated by cells of the neurovascular unit (Wareham and Calkins, 2020). Astrocytes are central to this process; each retinal astrocyte interfaces with both blood vessels and RGC somata and axons, positioning them as key regulators of vascular-neuronal communication (Holden et al., 2023). Through expression of GLUT1 at perivascular endfeet, astrocytes mediate basal glucose uptake from the circulation and distribute metabolic substrates to neurons (Maher, 1995; Kacem et al., 1998; Kumagai, 1999). This function is particularly critical for RGCs distal to the vasculature, which depend on astrocyte-derived metabolites, including lactate generated from glucose (Tabernero et al., 2006).

Astrocytic glucose uptake is dynamically regulated by neuronal activity. Glutamate release from RGCs is taken up by astrocytes, triggering intracellular Ca^2+^ signaling and increased glucose uptake through enhanced GLUT1 expression (Bernardinelli et al., 2004). In parallel, astrocyte-astrocyte coupling via gap junctions modulates metabolic distribution across the astrocytic network (Sánchez-Alvarez et al., 2004, 2005). In the brain, astrocytic GLUT1 expression exhibits plasticity in response to physiological and pathological stimuli, including exercise, aging, and injury, with changes occurring prominently at vascular endfeet where glucose uptake is initiated (Rosenstein et al., 1994; Choeiri et al., 2005; Allen and Messier, 2013; Ding et al., 2013; Souza et al., 2015). Emerging evidence implicates cGMP signaling as a regulator of GLUT1 transcription (Tian et al., 2017). Since dysfunctional cGMP signaling leads to aberrant retinal astrocyte morphology and RGC degeneration with age (Holden et al., 2022; Bossardet et al., 2026), understanding how cGMP may influence astrocyte-blood vessel connectivity may uncover novel pathways integral to neurovascular function in the retina. Here, we sought to explore how cGMP signaling modulates astrocytic GLUT1 distribution and its relationship to the retinal vasculature. Using the G-MORF mouse model which captures full membranous morphology of single retinal astrocytes (Holden et al., 2023), we show that acute elevation of cGMP signaling increases the coverage of individual astrocytes and enhances association with the retinal vasculature compared with vehicle-injected controls. Furthermore, 8-Br-cGMP promotes the enhancement of GLUT1 to astrocyte perivascular endfeet without altering its overall levels. Together, these findings suggest that cGMP signaling contributes to structural and molecular remodeling of astrocytes at the neurovascular interface, which may have implications for metabolic exchange in the retina.

## 2 Methods

### 2.1 Animals

**Figure 1:**
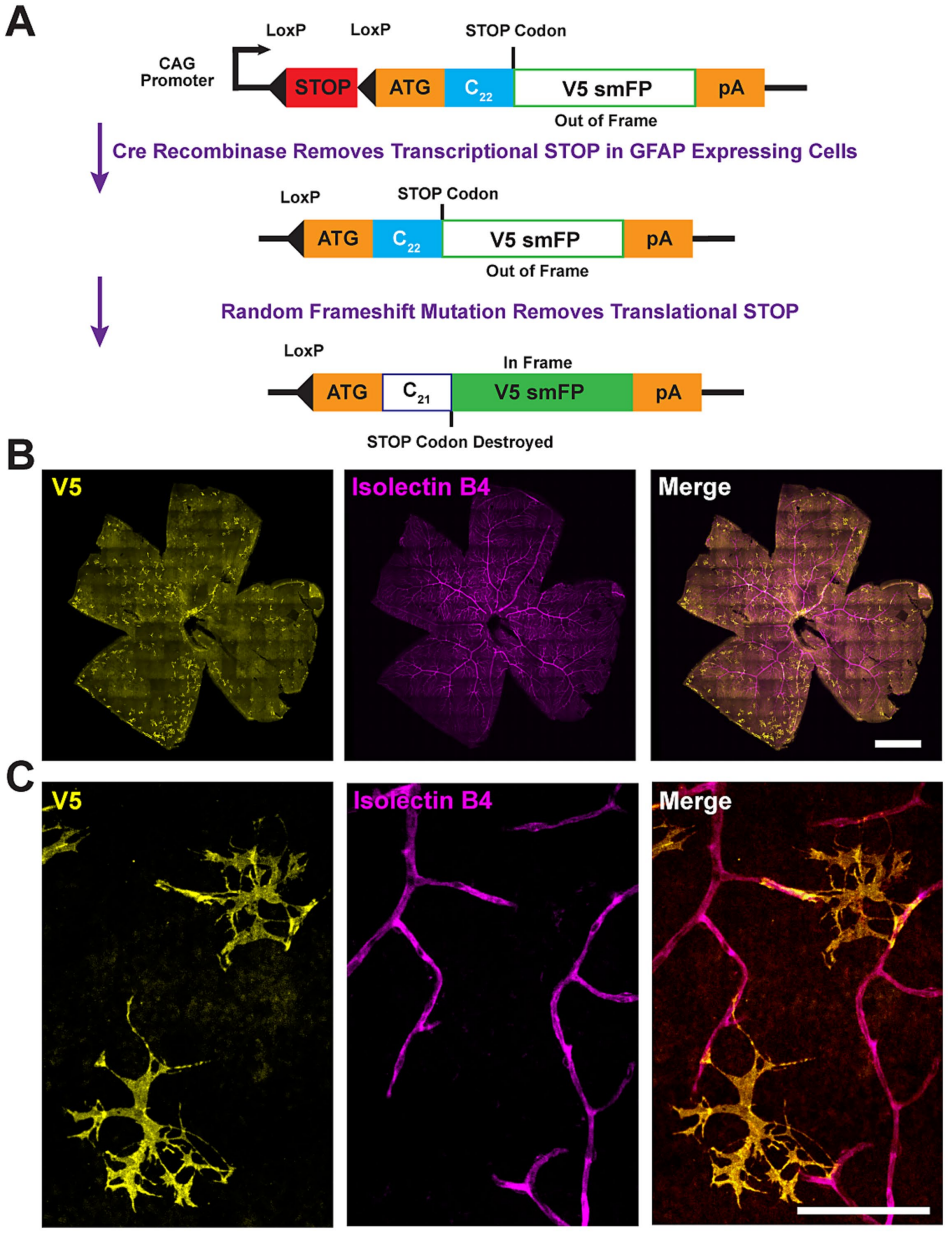
G-MORF mouse model. **(A)** The G-MORF system incorporates both transcriptional and translational STOP elements that prevent expression of the spaghetti monster fluorescent reporter until they are excised. Activation is achieved through a combination of stochastic mutation and Cre recombinase–mediated recombination. The model was generated by crossing MORF3 mice with GFAP-Cre 77.6 mice. **(B)** V5 immunolabeling in G-MORF retinas is sparse and yields largely isolated cells. Representative image of a wholemount retina labeled for V5 and GFAP (scale bar = 1 mm). **(C)** Representative examples of three GFAP-positive retinal astrocytes, each well separated and displaying complete membranous morphology (scale bar = 100 μm).

All animal experiments were conducted in accordance with the National Institutes of Health Guide for the Care and Use of Laboratory Animals and approved by the Vanderbilt University Medical Center IACUC (Protocol #M2300049). Animals were housed under a 12-h light/dark cycle with ad libitum access to food and water. Male and female G-MORF mice were generated by crossing Cre 77.6 mice (Jackson Laboratories, #024098) with MORF3 mice (Jackson Laboratories, #035403) as previously described (Holden et al., 2023); all mice were aged between 16 and 18 weeks. The G-MORF model expresses a membrane-targeted spaghetti monster fluorescent protein (smFP) containing 20 V5 epitopes, driven by a farnesylation sequence (Hancock et al., 1991). smFP expression requires a stochastic developmental frameshift event, resulting in sparse labeling (~1%) of retinal astrocytes. This enables clear resolution of individual cells, with ~75–150 well-separated, membrane-labeled astrocytes per retina (Figure 1). The smFP reporter is not intrinsically fluorescent and detection relies on immunohistochemistry as described below.

### 2.2 Study design and pharmacological intervention

Mice were sedated with isoflurane anesthesia (2.5%) and intravitreally injected bilaterally with 2 μL 8-Br-cGMP (Cat No. B1381; Sigma Aldrich) diluted in sterile 1x PBS to a final concentration of 200 μM or an equal volume of sterile 1x PBS (vehicle). After injection, mice returned to the mouse facility for 48 h before sacrifice. A 48-h post-injection timepoint was selected to assess early effects of acute cGMP elevation on astrocyte morphology and GLUT1 localization. This window allows sufficient time for signaling-dependent cytoskeletal remodeling and redistribution of membrane-associated proteins, while limiting the likelihood of longer-term secondary changes such as vascular remodeling, inflammatory responses, or neurodegeneration. Thus, the 48-h timepoint was intended to capture acute astrocyte responses to cGMP signaling at the neurovascular interface rather than chronic tissue adaptation.

### 2.3 Whole retina immunohistochemistry

Mice were sacrificed through intraperitoneal pentobarbital injection followed by transcardial perfusion of 1x PBS. Eyes were immediately enucleated and the retinas dissected fresh. Retinas were then fixed in 4% paraformaldehyde for 30 min at room temperature before washing in 1x PBS. Wholemount retinas were transitioned through a sucrose gradient (20%–30%) to stabilize the tissue for immunohistological staining. The retinas were then frozen at −80 °C in 30% sucrose and thawed at room temperature for a total of three freeze–thaw cycles to enhance antibody penetration. Samples were washed three times in 1x PBS then transferred to a blocking solution containing 5% normal donkey serum and 0.1% Triton-X 100 diluted in 1x PBS for 2 h at room temperature with gentle agitation. Retinas were incubated for 4 days at 4 °C in primary antibodies against V5 Tag (goat 1:500; Abcam ab95038), GLUT1 (rabbit 1:500; Abcam ab115730), and IB4 (647 conjugate 1:500; Invitrogen 132,450). Following primary incubation, retinas were washed three times in 1x PBS with gentle shaking and incubated for 2 h at room temperature in secondary antibody solution containing Alexa Fluor 488 anti-goat (1:200; Jackson ImmunoResearch) and Alexa Fluor 555 anti-rabbit (1:200; Jackson ImmunoResearch). Finally, retinas were washed three times in 1x PBS then mounted with DAPI fluoromount-G (Southern Biotech; Cat No. 0100–20), cover-slipped, and sealed with nail polish prior to imaging.

### 2.4 Fluorescent imaging

Images were acquired using a Nikon Ti-E Spinning Disk confocal microscope. A 20x objective was used to generate a montage of the entire retina, and the focus surface tool was utilized to determine the optimal Z position across the tissue. Based on coordinates obtained from the montage, individual astrocytes were then imaged at 60x magnification. Z-stacks of equal thickness were collected with a step size of 0.3 μm. Astrocytes located in clusters or overlapping with Müller glia were excluded from imaging and analysis. This process was repeated until all individual astrocytes within each retina had been imaged.

### 2.5 Single cell astrocyte and vessel analysis

Confocal images were processed in a blinded manner (i.e., users unaware of animal groups) and ImageJ was used to measure distance between astrocytes and blood vessels, the total GLUT expression in astrocytes, and co-localization of astrocyte GLUT with blood vessels in the retina. Background fluorescence and non-specific signal were removed via the Automatic Otsu Threshold function in ImageJ and manually by the Paintbrush and Freehand Selection tools. To restrict GLUT expression to the astrocyte, the binarized astrocyte Z-stack was added to the ROI manager and applied to the GLUT channel using the Clear Outside function to remove any signal outside the astrocyte boundary. Segmented images were then saved and analyzed with a custom Python script. Specifically, a three-dimensional distance map was generated using the fast-marching method implemented in scikit-fmm^1^. This approach calculates the shortest distance from each point within an astrocyte to the nearest point on a blood vessel, allowing us to assess how closely astrocytes and vessels are positioned in space. Using these processed images and distances we calculated several measurements. Total astrocyte and vessel area were determined as the total sum of pixels in the astrocyte and vessel regions, respectively. The area of overlap between astrocytes and vessels was calculated as the total sum of pixels in the astrocyte where distance from vessel is equal to zero. The percentage of GLUT signal that overlaps with blood vessels was measured by dividing the total sum of pixels of astrocyte-associated GLUT overlapping with vessel (where distance equals zero) by the total sum of pixels of GLUT in the entire astrocyte. These measurements can additionally be described by the equations below:


Area of Astrocyte=Total Count of Astrocyte Pixels

Area of Vessel=Total Count of Vessel Pixels

Area ofAstrocyte Overlapping with Vessel=Total Count of Pixels sharedbyAstrocyte and Vessel

$$\text{Normalized GLUT}1\;\text{Intensity}=\frac{\text{Total GLUT}1\;}{\text{Area ofAstrocyte}}$$

### 2.6 Statistical analyses

All data were tested for normality in GraphPad Prism 10 using the D’Agostino–Pearson test to determine the appropriate statistical approach. In all cases, datasets failed the normality criterion; accordingly, for comparisons between two groups, non-parametric Mann–Whitney tests were used and Brown–Forsythe tests were used to compare variance between groups. Astrocytes were analyzed at the single-cell level to capture biological heterogeneity within the retinal astrocyte population; collapsing measurements to a per-animal mean or median can lead to a reduction in the biological signal of interest. Because astrocytes were sampled within animals, individual cells are not fully independent observations and pooled single-cell comparisons should be interpreted in the context of within-animal nesting. We performed several animal-level analyses; variance was compared both at the single-cell level and per animal using Brown–Forsythe tests and the coefficient of variation (CV) was also calculated for each individual animal and compared between groups using a Mann–Whitney test. The number of animals per group is indicated in the figures. A total of 227 astrocytes were analyzed in vehicle-treated retinas and 260 astrocytes in cGMP-treated retinas.

## 3 Results

### 3.1 Retinal astrocyte size increases following pharmacological cGMP pathway activation

**Figure 2:**
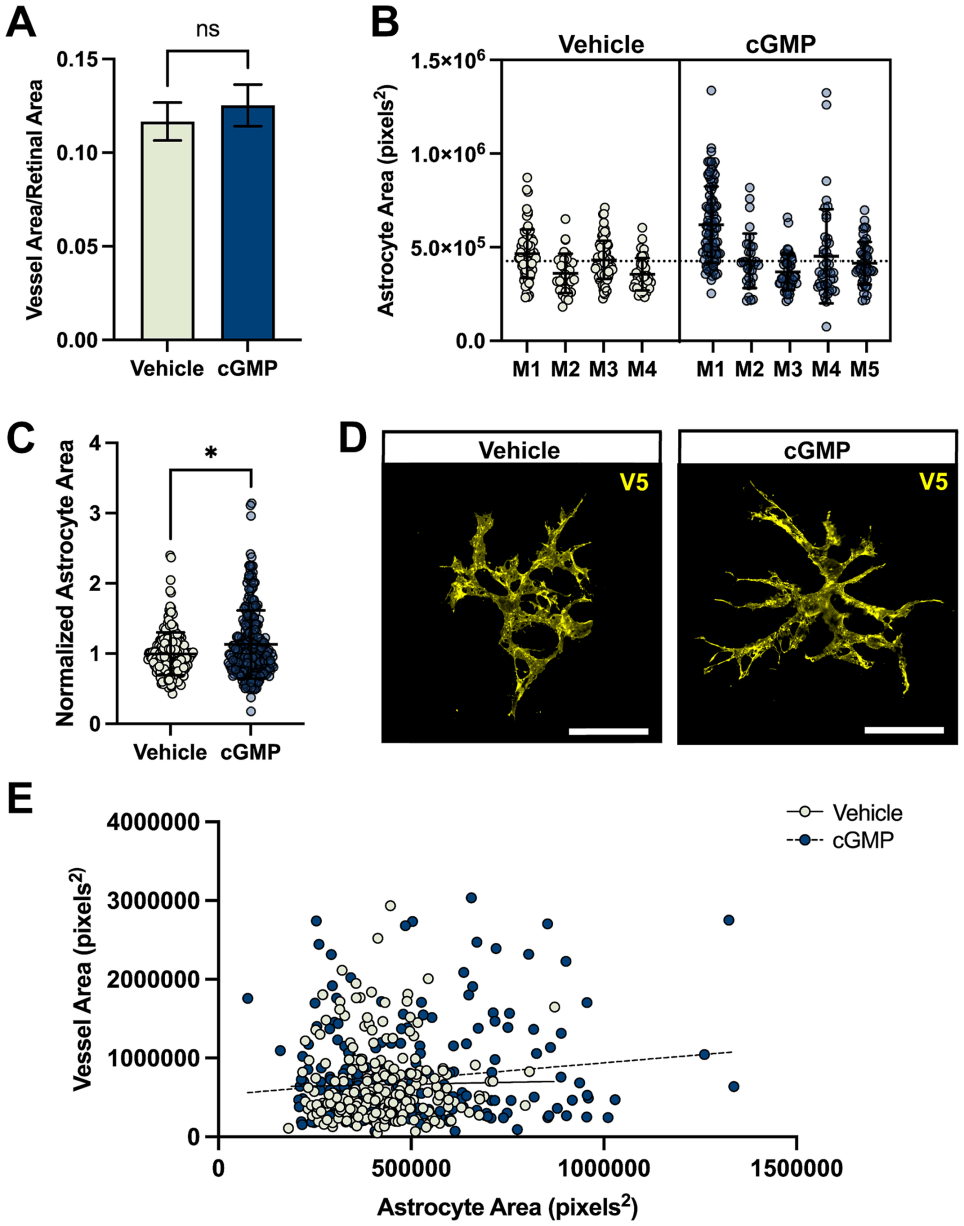
Astrocyte area increases with cGMP. **(A)** Total retinal vessel area normalized to retinal area did not increase with cGMP injection (*p* = 0.628). **(B)** Greater variance in astrocyte area observed in mice in the cGMP-injected group (dotted line = vehicle population mean = 418,610 pixels^2^; retinal astrocytes from *n* = 4 vehicle and *n* = 5 cGMP mice). **(C)** Astrocyte area increased after cGMP injection compared to vehicle-injected controls (*p* = 0.012). **(D)** Representative confocal micrographs of V5-expressing astrocytes in the retina of vehicle- and cGMP-injected animals, scale = 50 μm. **(E)** Astrocyte membranous area vs. local vessel pixel area in vehicle- and cGMP-injected animals show no significantly different correlation (*p* = 0.263). *T*-test = Mann–Whitney non-parametric test.

To resolve fine changes in single astrocyte morphology, we used the G-MORF transgenic mouse which enables full membranous visualization of isolated astrocytes across the retina, following a single intravitreal injection of either the membrane-permeable cGMP analog 8-Br-cGMP or vehicle (Figure 1). Given the well-established vasodilatory effects of cGMP (Lehners et al., 2018), we first determined whether treatment altered overall retinal vascular architecture. At 48 h post-injection, total retinal vessel area was unchanged between cGMP- and vehicle-treated groups (Figure 2A; *p* = 0.628), indicating that acute cGMP elevation does not globally remodel the retinal vasculature in the short term.

Astrocytes from cGMP-treated retinas exhibited greater variability in size, assessed by their coverage area, compared to controls (Figure 2B). We next quantified variance in astrocyte area at both the single-cell and per-animal levels. Variability was significantly increased in cGMP-treated retinas at the single-cell level (Brown–Forsythe test, *p* < 0.0001), indicating a broader distribution of astrocyte responses. This effect persisted when variance was calculated per animal (Brown–Forsythe test, *p* = 0.01), demonstrating that the increased variability observed was consistent across biological replicates and not driven by outliers. Because variance scales with the mean and the number of astrocytes sampled varied across animals, we calculated the coefficient of variation (CV) for each animal to assess relative variability. CV remained significantly higher in cGMP-treated animals (Mann–Whitney test, *p* = 0.03), indicating that increased heterogeneity is not solely attributable to changes in mean magnitude. Treatment with cGMP resulted in a significant increase (+13.5%) in astrocyte area compared to vehicle (Figure 2B,C; *p* = 0.012), demonstrating that astrocytes undergo morphological expansion in response to elevated cGMP signaling. Together, these findings demonstrate that cGMP signaling increases both absolute and relative variability in astrocyte responses, consistent with a more heterogeneous morphological response across the population. Representative confocal images illustrating these changes are shown in Figure 2D.

Because astrocyte morphology and proximity to blood vessels can influence vascular tone (Gordon et al., 2011; MacVicar and Newman, 2015; Endo et al., 2022), we next asked whether the observed increase in astrocyte size was associated with local changes in vessel area. Single-cell analysis revealed no significant correlation between astrocyte area and the area of adjacent vessels (Figure 2D; *p* = 0.263), suggesting that astrocyte expansion occurs independently of local vascular area. Together, these data indicate that elevated cGMP induces astrocyte morphological remodeling without altering overall vascular architecture or coupling astrocyte size to vessel caliber.

### 3.2 Pharmacological cGMP pathway activation increases astrocyte coverage of vessels

**Figure 3:**
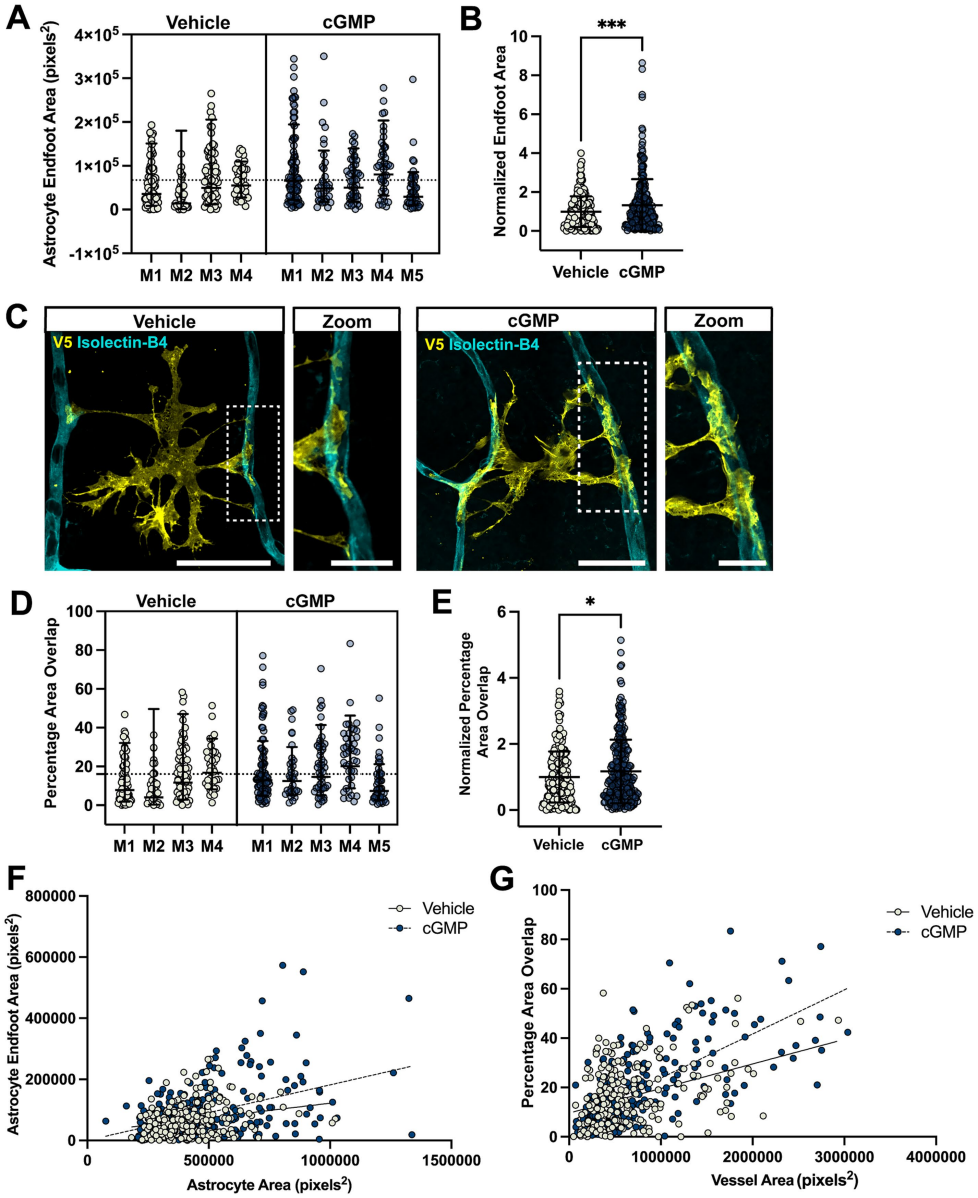
Astrocyte endfoot coverage of vessels increases with 8-Br-cGMP treatment. **(A)** Greater variance in astrocyte endfoot area was observed in mice in the cGMP-injected group (dotted line = vehicle population mean at 67228 pixels^2^). **(B)** Astrocyte endfoot area was increased by cGMP injection (*p* = 0.001). **(C)** Representative confocal micrographs of astrocytes (V5; yellow) and blood vessels (Isolectin-B4; cyan) in the retina of vehicle- and cGMP-injected animals (scale bar = 50 μm; 25 μm in zoomed image). **(D)** Greater variance in the percentage area of astrocyte overlap with vessels was observed in mice in the cGMP-injected group (dotted line = vehicle population mean at 16.05%). **(E)** The mean normalized percentage of each astrocyte area overlapping with a vessel was increased after cGMP injection (*p* = 0.03). **(F)** There was a greater correlation between increased astrocyte area and endfoot overlap with blood vessels in the cGMP-injected group compared to vehicle (*R*^2^ = 0.177 for cGMP vs. 0.05 for vehicle). The difference between the slopes was significant *p* = 0.04. **(G)** There was a greater correlation between increased local vessel area and percentage endfoot area overlap in the cGMP-injected group compared to vehicle (*R*^2^ = 0.461 for cGMP vs. 0.144 for vehicle). The difference between the slopes was significant (*p* = 0.0001). Individual animal data corresponding to F, and G shown in Supplementary Figure S1. All *T*-tests are Mann–Whitney non-parametric statistical tests.

To determine how cGMP influences astrocyte-vascular interactions, we quantified the extent of astrocyte endfoot coverage of blood vessels following vehicle or cGMP injection (Figure 3). Astrocytes in cGMP-treated retinas exhibited increased variability in endfoot area compared to vehicle (Figure 3A), suggesting a more heterogeneous structural response to cGMP signaling. Consistent with this finding, 8-Br-cGMP treatment resulted in a 33.24% increase in the area of astrocyte overlap (i.e., endfeet) with proximal vessels compared to vehicle (Figure 3B; *p* = 0.001), indicating enhanced astrocyte-vascular contact, as shown in representative confocal micrographs (Figure 3C). Although variance in the dataset at the single-cell level was significantly increased in cGMP-treated retinas (Brown–Forsythe test, *p* < 0.0001) and between animals (Brown–Forsythe test, *p =* 0.04), the coefficient of variation was not significantly different between groups (Mann–Whitney test, *p* > 0.1). These findings indicate that increased variability in endfoot overlap is largely attributable to changes in overall magnitude rather than an increase in relative heterogeneity. Taken together, these findings suggest that cGMP increases astrocyte endfoot-vessel overlap in a uniform manner.

Since astrocyte size increased following 8-Br-cGMP treatment, we next assessed whether increased overlap simply reflected larger astrocytes or a specific expansion of vascular contact. Unlike endfoot area, cGMP-treated astrocytes did not display increased variability in percent overlap compared to controls (Brown–Forsythe, *p* = 0.4, Figure 3D). The mean percentage of astrocyte area contacting vessels was significantly increased following 8-Br-cGMP treatment (Figure 3E; *p* = 0.03), indicating enhanced astrocyte-vascular association. To determine whether these changes reflected coordinated remodeling of astrocytes in relation to vascular structure, we examined the relationship between astrocyte size and endfoot overlap. In cGMP-treated retinas, this relationship was modestly strengthened compared to vehicle (*R*^2^ = 0.177 for cGMP vs. 0.05 for vehicle), with a significant difference in slope between groups (Figure 3F; *p* = 0.04). These results suggest that astrocyte expansion is associated with increased vascular contact under elevated cGMP conditions. Similarly, analysis of the relationship between local vessel area and astrocyte overlap revealed a difference in scaling between groups (Figure 3G). While both groups exhibited association between vessel size and astrocyte coverage, the relationship was significantly stronger with 8-Br-cGMP treatment (*R*^2^ = 0.461 for cGMP vs. *R*^2^ = 0.144 for vehicle; difference between the slopes *p* = 0.0001). Thus, with elevated cGMP astrocytes adjust their endfoot coverage in relation to vessel size. Note, that relationships hold within individual animals and are not driven by between-animal differences (Supplementary Figure S1). Together, these data demonstrate that cGMP signaling enhances astrocyte-vascular association by promoting both increased physical contact and improved scaling of endfoot coverage with vessel architecture.

### 3.3 Pharmacological cGMP pathway activation leads to astrocyte redistribution of GLUT1

**Figure 4:**
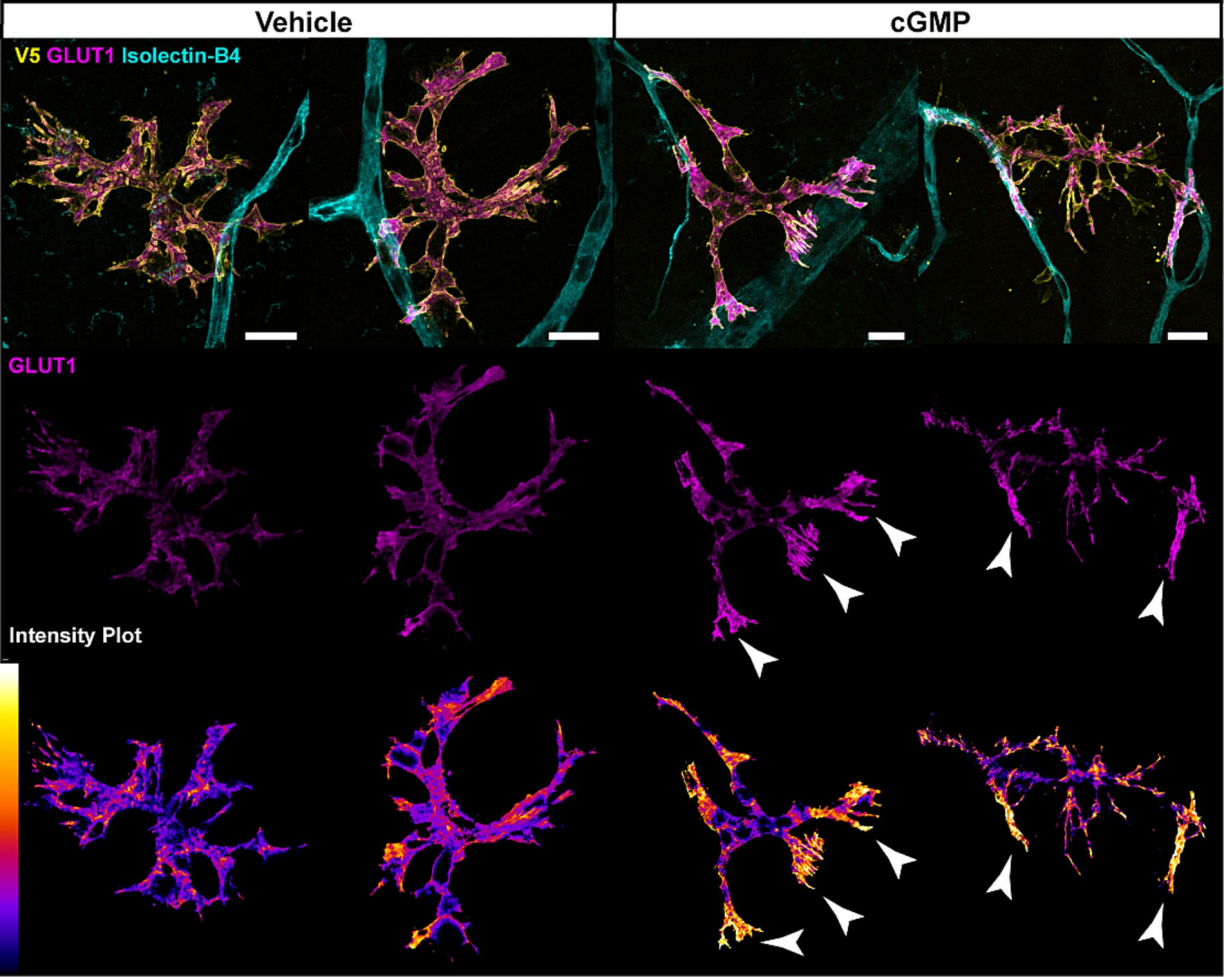
cGMP enhances GLUT1 localization at astrocyte endfeet. Representative confocal images of retinal astrocytes from vehicle- and cGMP-treated groups. Astrocyte morphology is visualized by V5 labeling (yellow), vasculature by isolectin-B4 (cyan), and GLUT1 by immunolabeling (magenta). For visualization purposes, V5 signal intensity was adjusted to emphasize astrocyte boundaries and facilitate assessment of GLUT1 localization. In cGMP-treated retinas, GLUT1 signal is enriched at astrocyte endfeet in contact with the vasculature (white arrows). This redistribution is further illustrated by GLUT1 intensity maps, where higher signal intensity is represented by white and lower intensity by purple/black. Scale bars = 20 μm.

GLUT1 is a primary mediator of glucose uptake at the cell membrane, and its expression and subcellular localization have been linked to cGMP signaling (Kc et al., 2005; Tian et al., 2017). To determine whether cGMP influences GLUT1 distribution in retinal astrocytes, we examined its localization following cGMP elevation. Representative confocal micrographs are shown in Figure 4. Qualitatively, astrocytes from cGMP-treated retinas exhibited increased GLUT1 signal at astrocyte endfeet in contact with the vasculature compared to vehicle controls (white arrows; Figure 4), which is consistent with enhanced localization of GLUT1 to the astrocyte-vascular interface.

**Figure 5:**
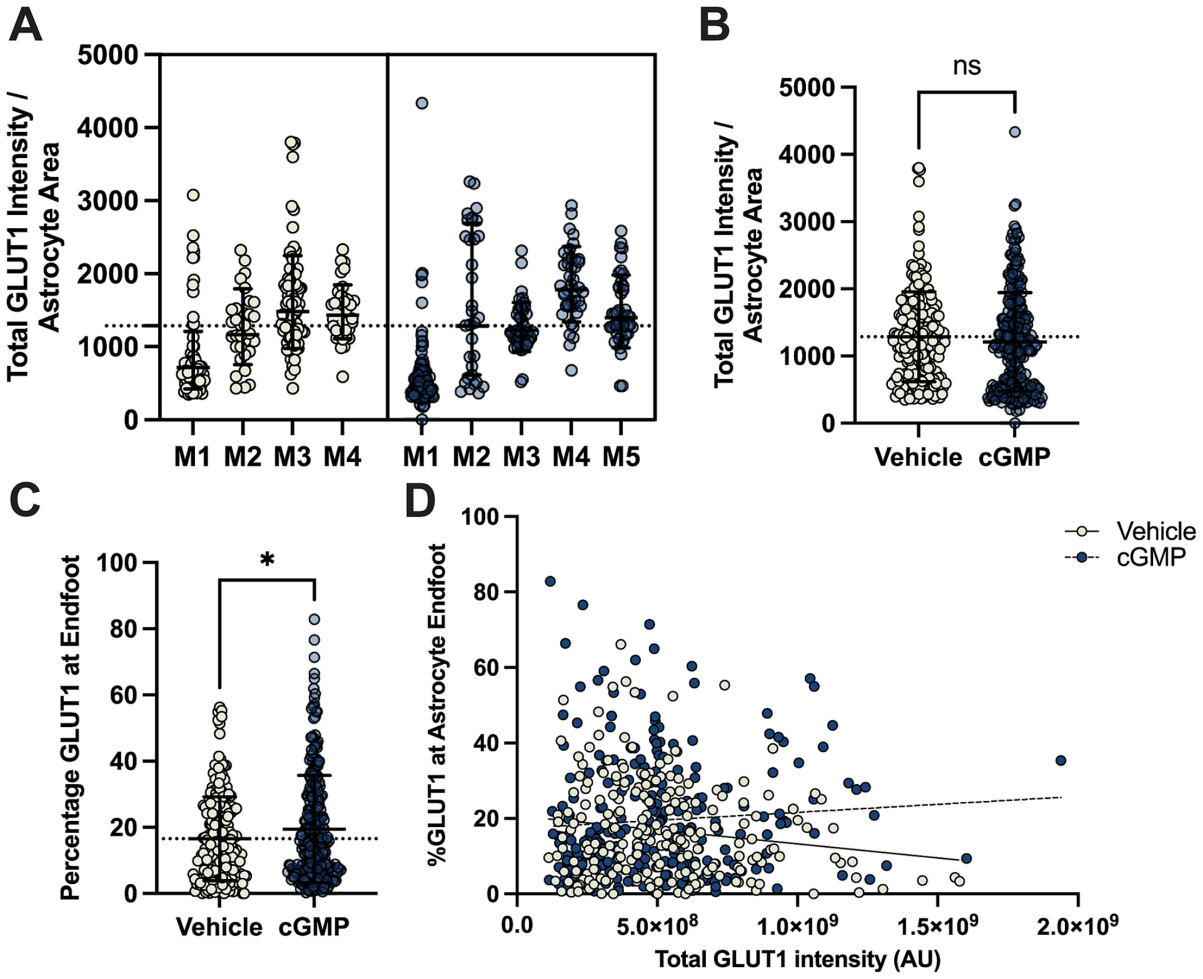
GLUT1 localization at astrocyte endfeet increases with cGMP. **(A)** Mean GLUT1 intensity does not significantly change between vehicle- and cGMP-injected groups (mean = 1,286 vs. 1,211). **(B)** Mean total GLUT1 intensity does not increase with cGMP (*p* = 0.273), dotted line indicates the vehicle group mean. **(C)** Mean percentage GLUT1 intensity at astrocyte endfeet increases with cGMP (+17.7%; *p* = 0.03), dotted line indicates the vehicle group mean. **(D)** Relationship between total cellular GLUT1 intensity and the percentage of GLUT1 localized to astrocyte endfeet. In both groups, the relationship is weak (*R*^2^ = 0.02 vs. 0.005), indicating that total GLUT1 levels do not strongly predict endfoot localization. However, the slopes differ significantly between vehicle- and cGMP-treated groups (*p* = 0.01), indicating that cGMP alters the relationship between total GLUT1 abundance and its subcellular distribution. Individual animal data corresponding to D shown in Supplementary Figure S1. All statistical comparisons were performed using Mann–Whitney non-parametric tests unless otherwise indicated. Each point represents a single astrocyte.

To confirm our qualitative observations in GLUT1 distribution we next quantitated astrocyte GLUT1 levels and distribution. First, we quantified total astrocytic GLUT1 intensity. Our results revealed similar levels between vehicle- and cGMP-treated groups when normalized for astrocyte size (Figure 5A,B; *p* = 0.273), indicating that cGMP does not globally increase GLUT1 protein abundance. We next asked whether cGMP influenced the spatial distribution of GLUT1 within individual astrocytes. Astrocytes from cGMP-treated retinas exhibited a 17.7% increase in GLUT1 localization to endfeet compared to controls (Figure 5C; *p* = 0.03), despite unchanged total GLUT1 levels. To further investigate this redistribution, we examined the relationship between total cellular GLUT1 and localization specifically to endfeet (Figure 5D). The relationship between total astrocyte GLUT1 and endfoot localization was weak in both vehicle- and cGMP-treated astrocytes (Figure 5D; *R*^2^ = 0.02 and 0.005, respectively). However, the slopes of these relationships differed significantly between groups, with 8-Br-cGMP treatment causing an increase in endfoot localization vs. a decrease in vehicle (*p* = 0.01). Note, that relationships hold within individual animals and are not driven by between-animal differences (Supplementary Figure S1). Thus, while total GLUT1 levels were not strongly predictive of endfoot localization in either condition, 8-Br-cGMP treatment significantly reversed the relationship between these variables. This indicates that cGMP may influence the distribution of GLUT1 within astrocytes independently of total protein levels.

## 4 Discussion

**Figure 6:**
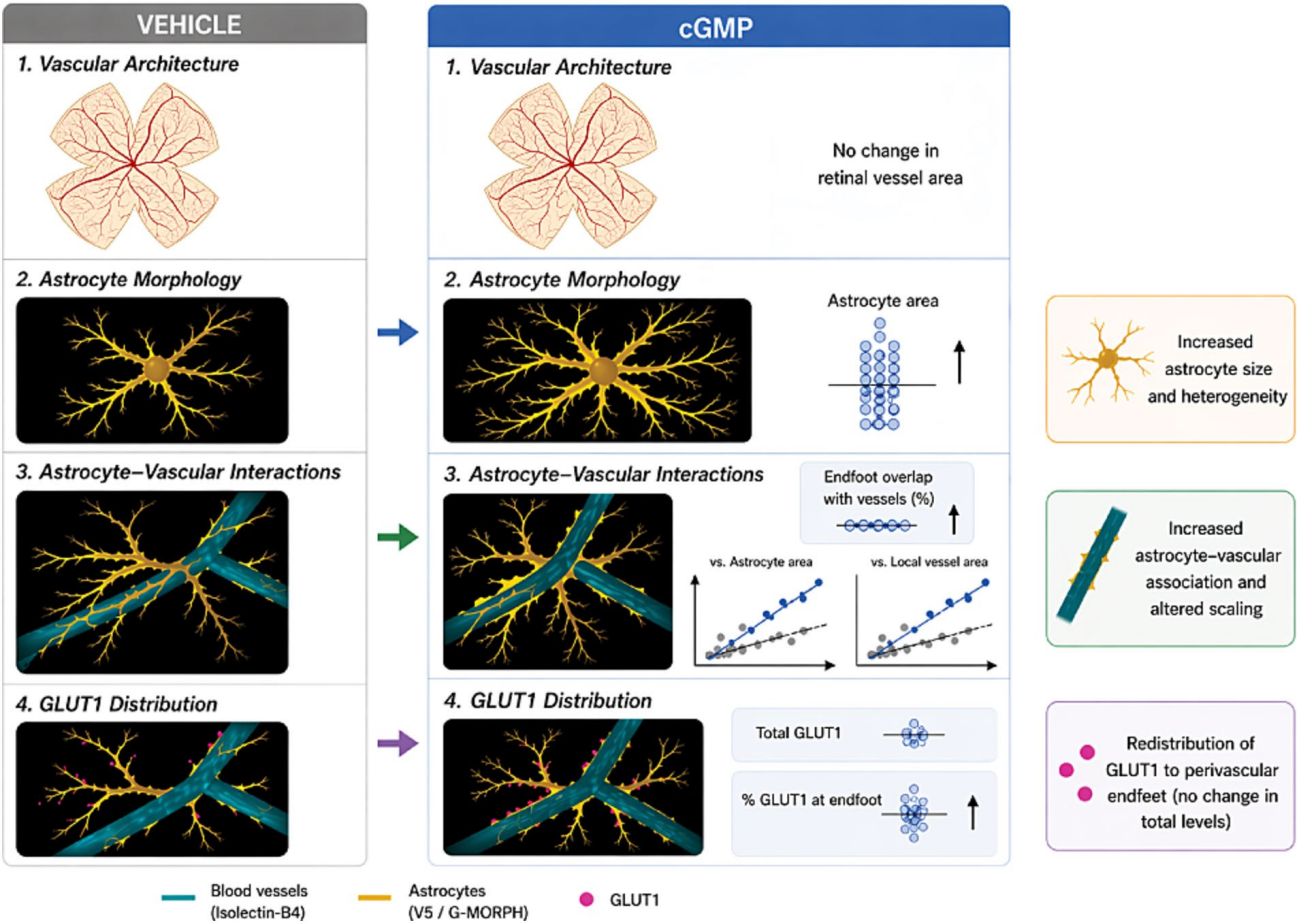
cGMP promotes astrocyte remodeling and perivascular enrichment of GLUT1 in the murine retina. Schematic summary comparing vehicle- and cGMP-treated retinas following intravitreal injection. cGMP does not alter overall retinal vascular architecture but is associated with increased astrocyte size and enhanced astrocyte-vascular association. cGMP also promotes redistribution of GLUT1 toward astrocyte endfeet at the vascular interface without changing total GLUT1 levels. Astrocytes (yellow), blood vessels (blue), and GLUT1 (pink).

In this study, we investigated how acute elevation of 8-Br-cGMP influences retinal astrocyte morphology, vascular interactions, and localization of the glucose transporter GLUT1. Our findings demonstrate that increased cGMP signaling induces structural remodeling of astrocytes characterized by increased membranous coverage (size), enhanced contact with the vasculature, and redistribution of GLUT1 to astrocyte endfeet, without altering overall vascular architecture, as summarized below (Figure 6).

Our key finding is that 8-Br-cGMP promotes astrocyte morphological expansion. This increase in astrocyte area occurred independently of changes in total or local vessel area, suggesting that astrocytes respond directly to cGMP signaling rather than secondarily to vascular remodeling. While cGMP is classically associated with regulating vascular tone (Derbyshire and Marletta, 2012), growing evidence indicates that cyclic nucleotide signaling also regulates glial structure and function in the retina and brain (Bender and Beavo, 2006; Friebe et al., 2020; Holden et al., 2022; Bossardet et al., 2026). Our data extend these observations by demonstrating that astrocytes in the retina undergo morphological remodeling in response to elevated cGMP signaling. Beyond changes in size, 8-Br-cGMP significantly enhanced astrocyte-vascular association. Astrocytes in 8-Br-cGMP-treated retinas exhibited increased absolute and proportional coverage of blood vessels, as well as improved scaling of endfoot coverage with vessel size. Astrocyte endfeet are key structural components of the neurovascular unit and play a central role in regulating blood flow, vascular stability, and exchange at the blood-retinal and blood–brain barriers (Abbott et al., 2006; Mathiisen et al., 2010; Iadecola, 2017). The strengthened relationship between vessel size and astrocyte coverage observed here suggests that increased cGMP signaling promotes a more coordinated structural interaction between astrocytes and the vasculature. Importantly, these effects were not fully explained by increased astrocyte size alone, indicating selective remodeling of astrocyte endfeet rather than uniform cellular expansion.

Consistent with these structural changes, we observed a redistribution of GLUT1 to astrocyte endfeet without an increase in total GLUT1 protein levels. Recent work defining the astrocyte endfoot proteome has revealed extensive enrichment of signaling and transport proteins, including GLUT1, highlighting the importance of subcellular localization in astrocyte function (Hill et al., 2025). GLUT1 is highly enriched at astrocyte endfeet and is essential for glucose transport across the blood–brain and blood-retinal barriers (Simpson et al., 2007; Winkler et al., 2015). The shift toward increased endfoot localization in 8-Br-cGMP-treated astrocytes suggests that cGMP signaling may regulate GLUT1 subcellular localization in addition to its transcriptional expression (Tian et al., 2017). This is consistent with prior work showing that GLUT1 localization and function are dynamically regulated in response to cellular signaling and metabolic demand (Vannucci et al., 1997; Mathiisen et al., 2010).

Several mechanisms may underlie the effects of 8-Br-cGMP on astrocyte morphology and GLUT1 localization we have observed. cGMP signaling through protein kinase G (PKG) is known to regulate cytoskeletal dynamics, including actin remodeling and focal adhesion turnover, which could drive the astrocyte morphological expansion observed here and promote extension of processes toward the vasculature (Francis et al., 2010). In addition, cGMP has been shown to influence astrocyte structure and reactivity *in vitro*, in part through modulation of RhoA/ROCK signaling and downstream effects on cellular contractility and process stability (Boran and Garcia, 2007). The selective enrichment of GLUT1 at astrocyte endfeet may reflect cGMP-dependent regulation of vesicular trafficking or membrane insertion, as cyclic nucleotide signaling pathways have been implicated in transporter membrane targeting in other cell types (Vinals et al., 1997; Bergemann et al., 2001). Finally, cGMP signaling may also act locally at the neurovascular interface downstream of endothelial-derived nitric oxide, providing spatially restricted cues that coordinate astrocyte process remodeling with vascular contact (Attwell et al., 2010; Iadecola, 2017).

Several limitations should be considered when interpreting the findings presented here. First, the study focuses on a single acute timepoint (48 h post-injection), and it remains unclear whether these effects are transient or sustained over longer durations. Second, the use of a pharmacological cGMP analog may not fully recapitulate endogenous cGMP signaling dynamics, including spatial compartmentalization and regulation by phosphodiesterases (Bender and Beavo, 2006). The dose of 8-Br-cGMP used in this study is higher than reported basal intracellular cGMP concentrations in retinal and CNS cells (~1–10 nM at rest which can rise to ~100–300 nM upon stimulation (Bender and Beavo, 2006; Friebe et al., 2020)). This was intentional to ensure penetration of 8-Br-cGMP through the vitreous body to the retina. The effective intracellular concentration reaching astrocytes is uncertain but likely lower than the injected dose. We also used a hydrolysis-resistant analogue insensitive to phosphodiesterase regulation to offset dilution by providing prolonged pathway activation. Future pharmacokinetic studies measuring retinal 8-Br-cGMP tissue concentrations following intravitreal delivery would greatly strengthen dose–response interpretations. Third, while the G-MORF model enables detailed morphological analysis, the functional consequences of astrocyte remodeling were not directly assessed. In particular, we did not measure glucose uptake, metabolic flux, or neuronal outcomes. GLUT1 localization was assessed using confocal immunohistochemistry, which, while sensitive to redistribution toward the perivascular endfoot compartment, does not provide the spatial resolution to distinguish between membrane-inserted and intracellular transporter pools. Future studies using super-resolution approaches (e.g., STORM microscopy) or cell-surface protein biotinylation on freshly isolated retinal preparations would enable definitive characterization of the membrane topology of redistributed GLUT1.

Finally, although our data are consistent with a direct effect of 8-Br-cGMP on astrocytes, we cannot exclude contributions from other retinal cell types. 8-Br-cGMP is a membrane-permeable, hydrolysis-resistant analogue that could potentially activate cGMP-dependent signaling in any responsive cell following intravitreal delivery, including retinal neurons (retinal ganglion cells, bipolar cells, amacrine cells, horizontal cells, and photoreceptors), endothelial cells, and pericytes. Neuronal cGMP signaling can modulate retinal activity and neurotransmitter release, raising the possibility that the observed astrocyte remodeling and GLUT1 redistribution represent indirect responses to altered neuronal or vascular cGMP activity. Future studies using cell-type-specific tools, such as astrocyte-targeted cGMP manipulation, will be required to definitively distinguish direct from indirect astrocyte effects (Iadecola, 2017; Friebe et al., 2020).

Our results redefine the functional consequences of cGMP-induced astrocyte remodeling. A key priority for future work will be direct measurement of glucose transport capacity following cGMP elevation. Approaches such as *ex vivo* two-photon glucose imaging with genetically encoded FRET sensors, or radiolabeled glucose uptake assays in isolated retinal preparations, will allow us to directly test whether the GLUT1 redistribution observed here translates into enhanced metabolic exchange at the astrocyte-vascular interface. Live imaging approaches could also be useful in assessing whether cGMP alters astrocyte-mediated regulation of blood flow or functional neurovascular coupling dynamics *in vivo*. An important direction for future work will be to examine whether the astrocyte remodeling effects of cGMP reported here are sex-dependent. Our prior work has demonstrated that disruption of cGMP signaling produces sexually dimorphic effects on RGC neurodegeneration (Bossardet et al., 2026) and astrocyte biology more broadly is increasingly recognized as sex-dependent, with differences in morphology, transcriptomics, and functional outputs documented across CNS regions in health and disease (Gozlan et al., 2024; Bortolanza et al., 2025). Notably, sex differences in astrocyte reactivity have been reported in the optic nerve where female astrocytes show a more pronounced transcriptional response than males during neuroinflammation (Tassoni et al., 2019). Given the known influence of gonadal hormones on cyclic nucleotide signaling and astrocyte physiology, disaggregating responses by sex may reveal important heterogeneity in how the neurovascular unit responds to cGMP elevation, with implications for understanding sex differences in retinal disease susceptibility and progression. While the present study included both male and female mice, it was not powered for sex-stratified comparisons, as such, any within-sex subgroup analysis would be severely underpowered and potentially misleading. Thus, a dedicated, sex-balanced study with adequate statistical power will be required to test whether male and female retinal astrocytes differ in their morphological and metabolic responses to cGMP elevation.

Finally, extending these findings to disease-relevant contexts generally such as aging or neurodegeneration may provide insight into whether modulation of cGMP signaling can restore astrocyte-vascular interactions under pathological conditions. Our early work indicates that dysfunctional cGMP signaling leads to degeneration of retinal ganglion cells with age in mice (Wareham et al., 2018; Holden et al., 2022; Bossardet et al., 2026) and that treatment with a phosphodiesterase inhibitor is neuroprotective (Wareham et al., 2018). Thus, defining the mechanisms underlying this protection is an important next step. Given that disruption of neurovascular coupling and glucose transport is a common feature of neurodegenerative diseases (Wareham et al., 2022), targeting pathways that enhance astrocyte-vascular interface function may represent a potential therapeutic strategy.

## 5 Conclusion

Taken together, these findings suggest that cGMP signaling coordinates structural and molecular remodeling of astrocytes within the retinal neurovascular unit. Rather than inducing broad changes in protein expression or vascular structure, increased cGMP signaling refines the spatial organization of astrocytes in a manner that could support more efficient exchange between blood vessels and neural tissue. While we did not directly assess metabolic function, the combined increase in astrocyte-vascular contact and enrichment of GLUT1 at endfeet is consistent with a potential role for cGMP in modulating metabolic coupling within the retina, aligning with broader evidence that astrocytes play a central role in coupling vascular supply to neuronal metabolic demand.

## Data Availability

The raw data supporting the conclusions of this article will be made available by the authors, without undue reservation.

## Footnotes

1
https://github.com/scikit-fmm/scikit-fmm

## Supplementary material

SUPPLEMENTARY FIGURE S1 Individual animal scatter plots for data corresponding to Figure 3F **(A,B)**, Figure 3G **(C,D)**, and Figure 5D **(E,F)**. Individual animal data indicated in figure legends.
